# 

**Published:** 1924-01

**Authors:** 


### HOSPITAL and HEALTH REVIEW

*Established 1886* *No.* 1870. *Vol. LXXIII.*

*New Series.* Vol. III. No. 28 *January,* 1924. Price Sixpence

CONTENTS.

| | PAGE |
|---|---|
| Pensions and Health | 1 |
| Insanity and Crime | 2 |
| Notes and Comments | 3 |
| Still Another Minister of Health—Progress of the King's Fund—Organising a Health Week—Pembrey and Paradox—Surgeon and Man of Letters—The Reformed Public-House—Foot and Mouth Disease—Fog as "Poison Gas"—A Home for Every Child—On Appointing a Professor—The Extinction of Leprosy—The Fatal Geyser—Is Twilight Sleep Waning?—The Prince of Wales and Sir Benjamin Brodie—Deaths from Poisoning—Can Syphilis be Stamped Out? | 3-6 |
| Hospital Men of Mark: Mr. E. W. Morris, C.B.E. | 7 |
| The Voluntary Hospitals of Manchester | 8 |
| A New Welfare Centre at Liverpool | 8 |
| The American Small Hospital | 9 |
| Hospital History of Cape Town | 10 |
| Hospital Progress and Finance | 11 |
| Clean Milk for Consumptives: Hospital Milk from A Garden City | 13 |
| Middlesex Hospital Reception | 15 |
| Festival Dinner of Poplar Hospital | 16 |
| The Hospitals' Handmaid | 16 |
| Hospital Sunday Fund | 16 |
| The Panel Fee Inquiry | 17 |
| Health Weeks in London: By Dr. Stella Churchill | 18 |
| The Truth About Poisoning with Furs | 19 |
| Princess Mary at Newcastle | 20 |
| King's College Hospital Pageant | 20 |
| East Suffolk and Ipswich Hospital | 21 |
| Hospital Saving Association | 21 |
| Centenary of Medical Missions | 22 |
| Reviews of Books | 23 |
| Correspondence | 27 |
| Surgical Aid Society | 28 |
| Organising a Health Week: A Prize of £100 | 29 |
| Echoes of the Month | 30 |
| Hospital and Health News | 32 |
| Answers to Correspondents | 34 |
| Recent Appointments | 34 |

